# The direct inhibitory effects of *Lactobacillus acidophilus*, a commensal urinary bacterium, on calcium oxalate stone development

**DOI:** 10.1186/s40168-024-01877-y

**Published:** 2024-09-17

**Authors:** Chadanat Noonin, Anantaya Putpim, Visith Thongboonkerd

**Affiliations:** 1 Medical Proteomics Unit, Research Department, Faculty of Medicine Siriraj Hospital, Mahidol University, Bangkok, 10700 Thailand; 2 Department of Dermatology, Faculty of Medicine Siriraj Hospital, Mahidol University, Bangkok, 10700 Thailand

**Keywords:** Adhesion, Aggregation, CaOx, Crystallization, *E. coli*, Growth, Stone prevention

## Abstract

**Background:**

*Lactobacillus acidophilus* is a commensal urinary bacterium found more abundantly in healthy individuals than in stone patients. Hence, it has been proposed to play an inhibitory role in kidney stone disease (KSD) but with unclear mechanisms. We therefore investigated the direct effects of *L. acidophilus* on calcium oxalate (CaOx) stone development compared with *Escherichia coli*, which is known to promote CaOx stone formation.

**Results:**

*L. acidophilus* at 1 × 10^3^ CFU/ml  significantly reduced the abundance of newly formed crystals, enlargement and aggregation of seeded crystals, and crystal adhesion on renal cell membranes. By contrast, *E. coli* at 1 × 10^3^ CFU/ml significantly enhanced crystal growth and aggregation but did not affect crystallization and crystal-cell adhesion. Oxalate consumption assay showed that neither *L. acidophilus* nor *E. coli* significantly reduced the remaining oxalate level after 1 − 3 h incubation. However, both of them adhered to CaOx crystals. Surface component detection revealed that only *L. acidophilus* expressed S-layer protein, whereas only *E. coli* exhibited flagella on their surfaces. Removal of *L. acidophilus* S-layer protein and *E. coli* flagella completely abolished the inhibitory and promoting effects of *L. acidophilus* and *E. coli*, respectively.

**Conclusions:**

*L. acidophilus* inhibits CaOx stone development by hampering crystallization, growth, aggregation and cell-adhesive ability of CaOx. By contrast, *E. coli* enhances CaOx stone development by promoting CaOx growth and aggregation. Their contradictory effects are most likely from differential surface components (i.e., S-layer protein on *L. acidophilus* and flagella on *E. coli*) not from oxalate-degrading ability.

Video Abstract

**Supplementary Information:**

The online version contains supplementary material available at 10.1186/s40168-024-01877-y.

## Introduction

Calcium oxalate (CaOx) is the main type of kidney stones found in over half of stone patients [1–4]. An early phase of CaOx stone development involves CaOx crystal formation as a result of supersaturation of urinary calcium and oxalate ions [5]. Once formed, the crystals can further enlarge, form aggregates and adhere to tubular epithelial cell membranes, resulting in stone development [4, 5]. As such, a high concentration of urinary calcium (hypercalciuria) or oxalate (hyperoxaluria) is a promoting factor that induces kidney stone disease (KSD) [5]. In addition, acidic urine, low urinary citrate concentration, high urinary uric acid concentration, and changes in urinary levels of other small molecules and/or macromolecules (proteins, glycosaminoglycans, etc.) can also induce KSD [5].

Apart from inorganic and organic molecules mentioned above, urinary microbiome also plays an important role in stone development [6, 7]. Bacteria that belong to the *Enterobacteriaceae* family are more abundant in the urinary microbiome of stone patients compared with that of healthy individuals [8, 9]. *Escherichia coli*, a member of the *Enterobacteriaceae* family, is the most abundant bacterial species found in kidney stone matrices [10] and can enhance CaOx growth and aggregation [11, 12]. Similarly, *Klebsiella pneumoniae*, another bacterium found in stone matrices can also aggravate CaOx growth and aggregation [10, 11].

Indeed, bacterial diversity in the urinary microbiome of healthy individuals is greater than that of stone patients [8, 13]. Several commensal bacteria have been identified in the urine of healthy individuals without urinary tract infection [14–16]. *Lactobacillus* is an abundant bacterial genus found in normal human urine [17, 18], especially in healthy females [15, 19], and enrichment of *Lactobacillus* spp. can distinguish healthy individuals from stone patients [8, 9, 18]. Examples of these *Lactobacillus* spp. include *L. acidophilus, L. crispatus, L. iners*, and *L. johnsonii*, which are found in the urine of healthy individuals [14, 15, 18]. *L. crispatus* and *L. iners* are more abundant in healthy individuals than in stone patients [18]. Additionally, *L. crispatus* and *L. johnsonii* are more abundant in female urine than in male urine [15]. Note that females generally have a lower prevalence of KSD [20, 21]. However, urinary bacterial species identified from different studies seem to be inconsistent (possibly due to different sample collection and identification methods) [22]. Despite the inconsistency, *L. acidophilus* is a common commensal bacterium found in the urine of both healthy males and healthy females [15]. On the other hand, some of the commensal bacteria, such as *Gardnerella, Lactobacillus* and *Prevotella* spp., are less abundant in the urine of stone patients [9, 13, 18, 23, 24].

It is therefore suggestive that the presence of some commensal bacteria in the urine is a protective factor against KSD [8, 18]. However, mechanisms underlying such protective effects are still unclear. Thus, the present study investigated the direct effects of *L. acidophilus*, a common urinary commensal bacterium, on CaOx stone development using various crystal assays compared with *E. coli*, which is known to promote CaOx stone formation [11, 12, 25]. Potential mechanisms underlying such direct effects were also explored.

## Materials and methods

### Bacterial culture

*L. acidophilus* (ATCC 314™) and *E. coli* (ATCC 25922™) (strain Seattle 1946) were from ATCC (Manassas, VA). The strain identity of both microbes was verified by whole-genome sequencing. *L. acidophilus* was cultured using *Lactobacillus* MRS broth (HiMedia Laboratories; Maharashtra, India). The culture was performed at 37 °C in an incubator supplied with 5% CO_2_. *E. coli* was cultured in LB broth (BD Biosciences; San Jose, CA) at 37 °C in a shaking incubator. The growth curve of each bacterium was constructed. Bacterial cells at the mid-log phase were collected by centrifugation at 6,000 × g for 10 min and washed with crystallization buffer (10 mM Tris–HCl and 90 mM NaCl, pH 7.4). After another centrifugation, bacterial cells were resuspended in the crystallization buffer. Since this study aimed to examine the effects of bacteria under normal (non-infection) condition, hence, the final concentration of bacteria used in almost all crystal assays was 1 × 10^3^ CFU/ml.

### Mammalian cell culture

Mouse inner medullary collecting duct (mIMCD-3) (ATCC) cells were cultured in Dulbecco's Modified Eagle Medium F12 (Gibco; Grand Island, NY) supplemented with 10% fetal bovine serum (Gibco) (heat-inactivated), 60 U/ml penicillin G (Sigma-Aldrich; St. Louis, MO) and 60 µg/ml streptomycin (Sigma-Aldrich) at 37 °C with 5% CO_2_.

### CaOx crystallization assay

The assay was performed following a protocol described previously [26, 27]. Briefly, 500 µl of 10 mM CaCl_2_·2H_2_O (Merck; Rahway, NJ) in crystallization buffer was added to each well of 24-well plate (Corning Inc.; Corning, NY) followed by *L. acidophilus* or *E. coli* (1 × 10^3^ CFU in 10 µl crystallization buffer). Thereafter, 500 µl of 1 mM Na_2_C_2_O_4_ (Sigma-Aldrich) in crystallization buffer was gradually added to each well. The mixture of CaCl_2_·2H_2_O and Na_2_C_2_O_4_ without bacteria served as the control. Formation of CaOx crystals was allowed by incubation at 25 °C for 1 h. The newly formed crystals were imaged under a phase-contrast inverted microscope (Eclipse Ti-S; Nikon; Tokyo, Japan). Crystal sizes were measured from all crystals in ≥ 15 random fields in each well using NIS Elements D software (v.4.11) (Nikon). Crystal abundance was then calculated as follows.

1 $$Crystal\ abundance ({\mu m}^{2}/field) = \sum Crystal\ sizes\ of\ all\ crystals\ in\ each\ field$$

### Fourier transform infrared (FTIR) spectroscopy

The chemical composition of CaOx crystals generated in this study was analyzed using FTIR spectroscopy (Nicolet 6700; Thermo Scientific Inc.; Waltham, MA) following a protocol described previously [28, 29]. The FTIR scan number was set at 32 with a resolution of 2 cm^−1^, and the FTIR spectra were collected at wavenumbers of 4000–700 cm^−1^. The obtained spectra were compared with the reference FTIR spectra in the “Kidney Stone Library—Basic” FTIR database.

### CaOx growth assay

The assay was performed following a protocol described previously [30, 31]. First, 500 µl of 10 mM CaCl_2_·2H_2_O in crystallization buffer was added to each well of 24-well plate followed by 500 µl of 1 mM Na_2_C_2_O_4_ in crystallization buffer. Formation of CaOx crystals was allowed by incubation at 25 °C for 1 h. At this time-point (T_0_), the obtained crystals were imaged under a Nikon Eclipse Ti-S microscope. *L. acidophilus* or *E. coli* (1 × 10^3^ CFU in 10 µl crystallization buffer) was then added to each well. The sample without bacteria served as the control. Enlargement of these seeded crystals was allowed by further incubation at 25 °C for 60 min. At this time-point (T_60_), the crystals were reimaged. Crystal sizes were measured from ≥ 100 crystals in ≥ 10 random fields in each well at both T_0_ and T_60_ using NIS Elements D software (v.4.11). Crystal growth was determined by Δ crystal size as follows.

2 $$\Delta\ crystal\ size \left({\upmu m}^{2}\right)= Average\ size\ at\ {T}_{60} \left({\upmu m}^{2}\right)- Average\ size\ at\ {T}_{0} ({\upmu m}^{2})$$

### CaOx aggregation assay

The assay was performed following a protocol described previously [12, 32]. First, 10 ml of 1 mM Na_2_C_2_O_4_ in crystallization buffer was gently added to 10 ml of 10 mM CaCl_2_·2H_2_O in crystallization buffer. The mixture was incubated at 25 °C overnight, and CaOx crystals were collected by centrifugation at 2,000 × g for 10 min, washed with methanol, and allowed to air-dry. The crystals (0.5 mg) were resuspended in 1 ml crystallization buffer in each well of 6-well plate (Corning Inc.) followed by *L. acidophilus* or *E. coli* (1 × 10^3^ CFU in 10 µl crystallization buffer). The sample without bacteria served as the control. Aggregation was allowed by incubation at 25 °C for 1 h in a shaking incubator (150 rpm) (Zhicheng; Shanghai, China). The obtained crystal aggregates were imaged under a Nikon Eclipse Ti-S microscope. Their number was counted from ≥ 15 random fields in each well.

### Crystal-cell adhesion assay

The assay was performed following a protocol described previously [33, 34]. The mIMDC-3 cells were plated in each well of 6-well plate (4 × 10^5^ cells/well) and allowed to grow for 48 h to form a monolayer. The culture medium was replaced by the fresh one containing 100 µg/ml CaOx crystals. *L. acidophilus* or *E. coli* (1 × 10^3^ CFU in 10 µl crystallization buffer) was then added to each well. The sample without bacteria served as the control. After 1-h incubation, the medium was discarded and the cell monolayer was washed with PBS to eliminate unadhered crystals. The remaining (adhered) crystals were then imaged under a Nikon Eclipse Ti-S microscope. Their number was counted from ≥ 15 random fields in each well.

### Oxalate consumption assay

The assay was performed following a protocol described previously [35]. *L. acidophilus* or *E. coli* at 1 × 10^3^, 1 × 10^4^ or 1 × 10^5^ CFU/ml in crystallization buffer was incubated with 0.5 mM Na_2_C_2_O_4_, whereas crystallization buffer containing 0.5 mM Na_2_C_2_O_4_ (without bacteria) served as the control. After 1-h incubation at 37 °C, bacterial cells were removed by centrifugation at 6,000 × g for 10 min, and the remaining oxalate concentration in the supernatant was determined by measuring its absorbance at λ214 nm using a UV–visible spectrophotometer (Analytik Jena AG; Jena, Germany) compared with a standard curve [35–37]. Sensitivity of oxalate measurement was determined by a standard method [38–40] applied to many recent works [41–43], with known oxalate concentrations at 0, 0.1, 0.2, 0.3, 0.4, 0.5, 0.6, 0.7 and 0.8 mM to create the standard curve. The sensitivity (k_A_) was calculated as follows.

3 $${k}_{A} (A.U./mM) = [Analyte^{\prime}s\ signal\ ({S}_{A}) (A.U.) - Reagent\ blank^{\prime}s\ signal ({S}_{Blank}) (A.U.)] / Analyte^{\prime}s\ concentration ({C}_{A}) (mM)$$

*where A.U.* = *Arbitrary (absorbance) unit.*

In addition, *L. acidophilus* or *E. coli* at 1 × 10^3^ CFU/ml was tested by this assay with 1-, 2- or 3-h incubation to examine whether a prolonged incubation affected the result. Moreover, *L. acidophilus* at 1 × 10^6^ or 1 × 10^7^ CFU/ml was also tested by this oxalate consumption assay with 1-h incubation to ensure that the assay provided a positive result when bacterial number increased.

### Bacteria-crystal binding assay

CaOx crystals (0.3 mg/ml in crystallization buffer) were mixed with *L. acidophilus* or *E. coli* (10^5^ CFU/ml in crystallization buffer) and incubated at 25 °C for 1 h on a rotator. The crystals were then collected by centrifugation at 2,000 × g for 10 min, washed twice with PBS, and fixed with methanol.

For qualitative analysis, the fixed crystals were incubated with 1 µg/ml Hoechst dye (Sigma-Aldrich) for 15 min to stain bacterial DNA. After centrifugation at 2,000 × g for 10 min, the supernatant was discarded and the crystals were resuspended in a mounting medium (15% glycerol in PBS) and mounted on a glass slide. The bacteria adhered to the crystals were then examined under a fluorescence microscope (Eclipse 80i; Nikon).

Quantitative analysis of the bacteria-bound crystals was done by flow cytometry. The crystals without bacterial incubation served as the control. The fixed crystals were incubated with 1 µg/ml propidium iodide (BD Biosciences) at 25 °C for 5 min to stain bacterial DNA. The data were analyzed by using a BD Accuri C6 flow cytometer (BD Biosciences).

### Detection of bacterial surface components

Immunofluorescence staining was performed to detect S-layer protein on bacterial surface. *L. acidophilus* or *E. coli* was fixed with 4% paraformaldehyde at 25 °C for 15 min, washed with PBS and incubated with 1% bovine serum albumin in PBS (blocking solution) for 1 h. The bacteria were then incubated with rabbit anti-S-layer protein polyclonal antibody (Biorbyt; Cambridge, UK) at 4 °C overnight, followed by Alexa Fluor546-conjugated anti-rabbit secondary antibody (Invitrogen; Waltham, MA) at 25 °C for 1 h. The immunofluorescence signal of the S-layer protein was detected under a Nikon Eclipse 80i fluorescence microscope.

To detect bacterial flagella, Gray’s flagella staining was performed as described previously [12, 44]. *L. acidophilus* or *E. coli* suspension was dropped on a glass slide and allowed to air-dry. The bacteria were incubated with iron tannate dye (Sigma-Aldrich) at 25 °C for 10 min, washed with distilled water, and incubated with carbol-fuchsin (Sigma-Aldrich) at 25 °C for 10 min. After rinsing with tap water, the stained samples were allowed to air-dry and imaged under a Nikon Eclipse 80i fluorescence microscope.

### Removal of *L. acidophilus *S-layer protein and *E. coli* flagella

S-layer protein was removed from *L. acidophilus* as described previously [45]. *L. acidophilus* cell pellet was resuspended in 5 M LiCl (Kemaus; New South Wales, Australia) (1 g wet weight of bacterial cell pellet per 5 ml of 5 M LiCl) and incubated in a shaking incubator (200 rpm) at 37 °C for 2 h. Bacterial cells were collected, washed three times with crystallization buffer, and subjected to immunofluorescence staining of S-layer protein and crystal assays as described above for the intact bacteria.

Flagella were removed from *E. coli* following a protocol reported previously [12]. *E. coli* cell pellet (from 10 ml culture at mid-log phase) was resuspended in 1 ml of 10 mM HEPES (Sigma-Aldrich), and the mixture pH was adjusted to 4.5 using 0.5 N acetic acid (RCI Labscan; Bangkok, Thailand). After 45-s incubation, 0.5 M KOH (AppliChem GmbH; Darmstadt, Germany) was added to the suspension until pH 7 was obtained. Bacterial cells were collected, washed three times with crystallization buffer, and subjected to Gray’s flagella staining and crystal assays as described above for the intact bacteria.

### Statistical analysis

All quantitative data were obtained from three experiments using independent biological replicates and are shown as mean ± SD. All datasets were tested for normal distribution before comparative analysis using either Kruskal–Wallis test followed by Dunn’s multiple comparisons (nonparametric) or One-way ANOVA followed by Tukey’s multiple comparisons (parametric). All *P* values reported herein were adjusted (for multiple comparisons), and the statistical significance was considered when adjusted *P* was < 0.05.

## Results

### Effects of bacteria on CaOx stone development

**Fig. 1:**
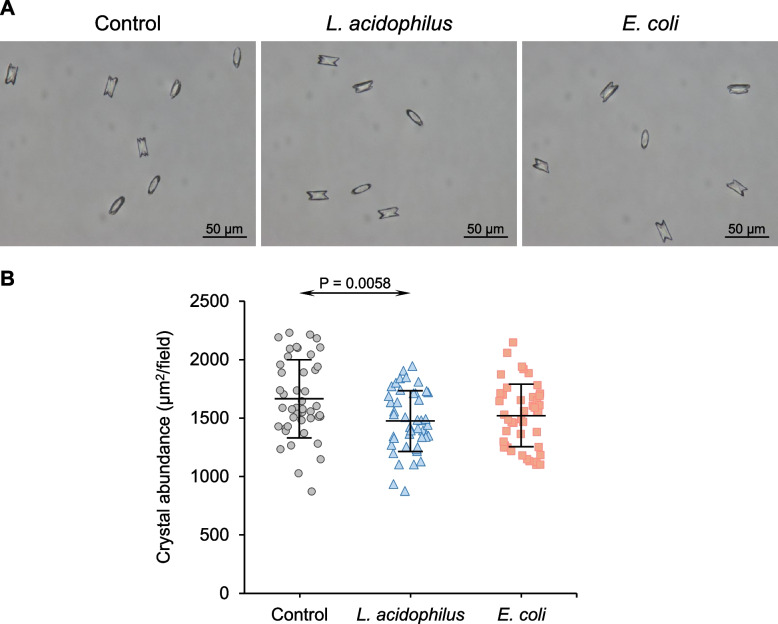
Effect of bacteria on CaOx crystal formation. Crystallization assay was performed without (control) or with 1 × 10^3^ CFU/ml of *L. acidophilus* or *E. coli*. **A** Representative images of CaOx crystals that were generated under different conditions. **B** Crystal abundance was calculated using *Formula I* (see Materials and methods) from ≥ 15 random fields in each sample. Quantitative data were obtained from three independent experiments using independent biological replicates. The error bar represents SD, and only a significant *P* value is indicated

Effects of bacteria on different steps of CaOx stone development were determined by using various CaOx crystal assays. The final concentration of bacteria used in these assays was 1 × 10^3^ CFU/ml to address the effects of bacteria under normal (non-infection) state. The crystal samples without bacteria served as the control. Crystallization assay revealed that *L. acidophilus* significantly reduced the abundance of newly formed crystals compared with the control (Fig. 1A and B). However, *E. coli* did not show a significant effect on CaOx crystal formation (Fig. 1A and B). The CaOx crystals generated in this study were analyzed for their chemical composition using FTIR spectroscopy. The data showed that our CaOx crystals (Supplementary Figure S1B) perfectly matched with CaOx monohydrate in the reference database (Supplementary Figure S1A).

**Fig. 2:**
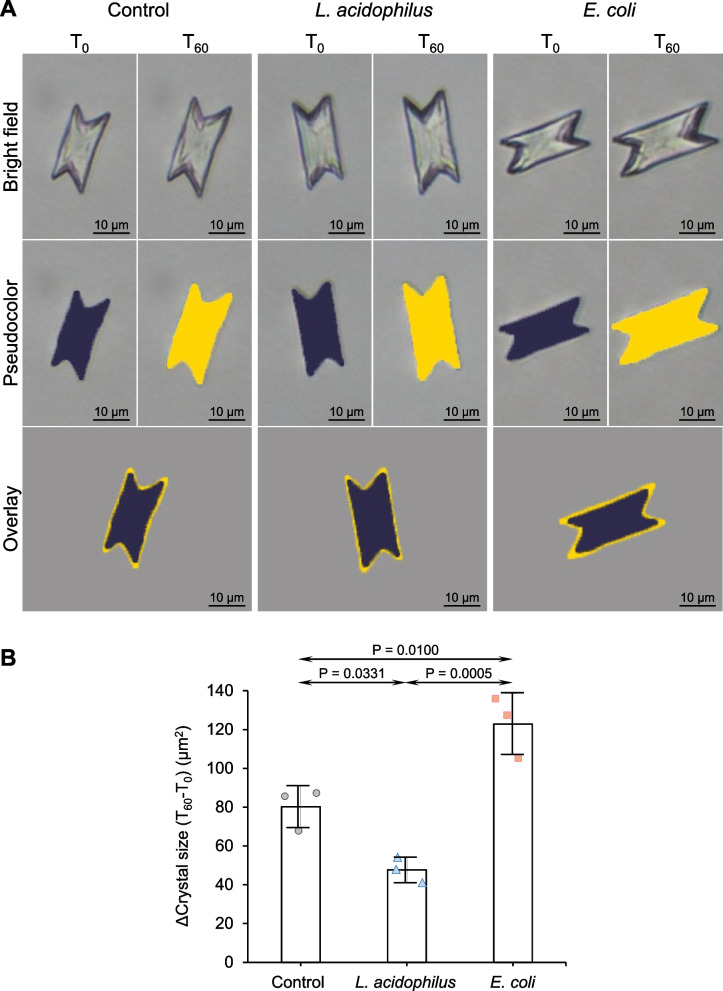
Effect of bacteria on CaOx enlargement. Crystal growth assay was performed without (control) or with 1 × 10^3^ CFU/ml of *L. acidophilus* or *E. coli*. **A** Representative images of CaOx crystals at T_0_ and T_60_ that were generated under different conditions. Pseudocolor and overlay views were created to enhance the visualization of differential sizes of crystals at T_0_ and T_60_. **B** Δ crystal size representing the growth of crystals was calculated using *Formula II* (see Materials and methods) from ≥ 100 crystals in ≥ 10 random fields in each sample. Quantitative data were obtained from three independent experiments using independent biological replicates. The error bar represents SD, and only significant *P* values are indicated

**Fig. 3:**
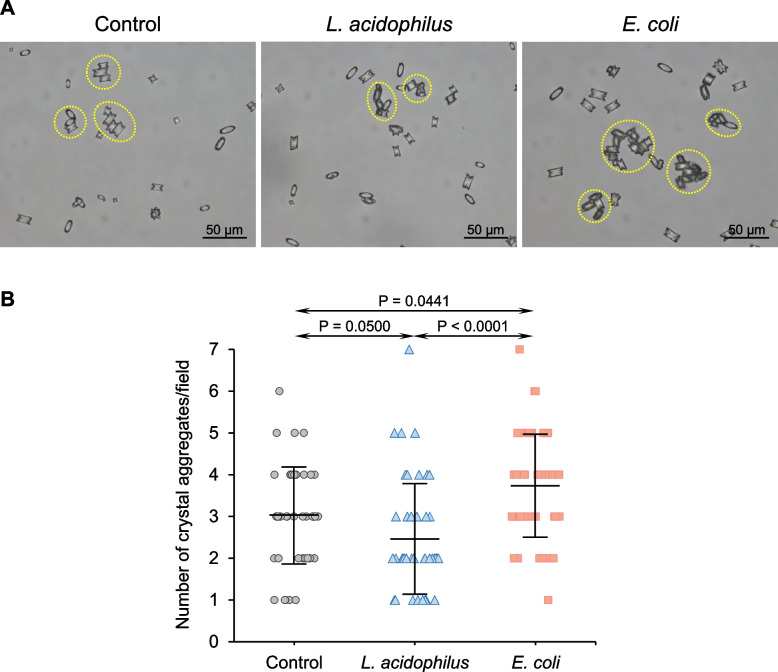
Effect of bacteria on CaOx aggregation. Crystal aggregation assay was performed without (control) or with 1 × 10^3^ CFU/ml of *L. acidophilus* or *E. coli*. **A** Representative images of CaOx aggregates (marked with dotted circles) that were generated under different conditions. **B** The aggregate number was counted from ≥ 15 random fields in each sample. Quantitative data were obtained from three independent experiments using independent biological replicates. The error bar represents SD, and only significant *P* values are indicated

**Fig. 4:**
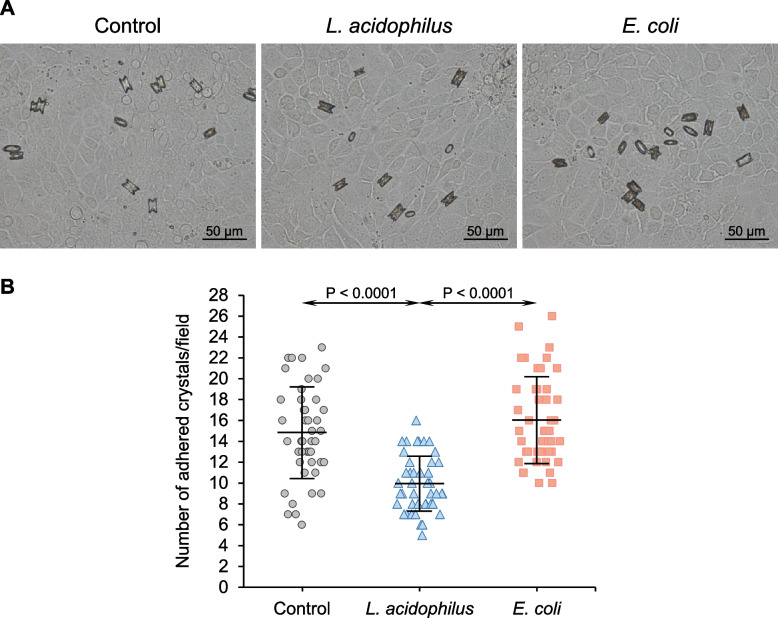
Effect of bacteria on CaOx crystal-cell adhesion. Crystal-cell adhesion assay was performed without (control) or with 1 × 10^3^ CFU/ml of *L. acidophilus* or *E. coli*. **A** Representative images of adherent CaOx crystals that remained on the cell monolayer under different conditions. **B** Number of the adhered crystals was counted from ≥ 15 random fields in each sample. Quantitative data were obtained from three independent experiments using independent biological replicates. The error bar represents SD, and only significant *P* values are indicated

Crystal growth assay demonstrated that *L. acidophilus* significantly reduced the enlargement of seeded crystals (Fig. 2A) as revealed by the decreased Δ crystal size compared with the control (Fig. 2B). Contrarily, *E. coli* significantly increased Δ crystal size compared with the control and *L. acidophilus* (Fig. 2A and B). The effects of these two bacteria on the aggregation of seeded crystals were also evaluated. The data showed that *L. acidophilus* significantly decreased the number of crystal aggregates compared with the control (Fig. 3A and B). By contrast, *E. coli* significantly increased the number of aggregates compared with the control and *L. acidophilus* (Fig. 3A and B). Similar to the crystallization assay, the crystal-cell adhesion assay demonstrated a significant reduction in the number of adherent crystals on the cell monolayer (Fig. 4A and B). However, *E. coli* did not show a significant effect on crystal binding to the cells (Fig. 4A and B).

These data indicated that *L. acidophilus* hampered CaOx crystal formation, enlargement, aggregation and adhesion to the cells. On the contrary, *E. coli* enhanced CaOx crystal enlargement and aggregation.

### Determination of oxalate consumption by bacteria

**Fig. 5:**
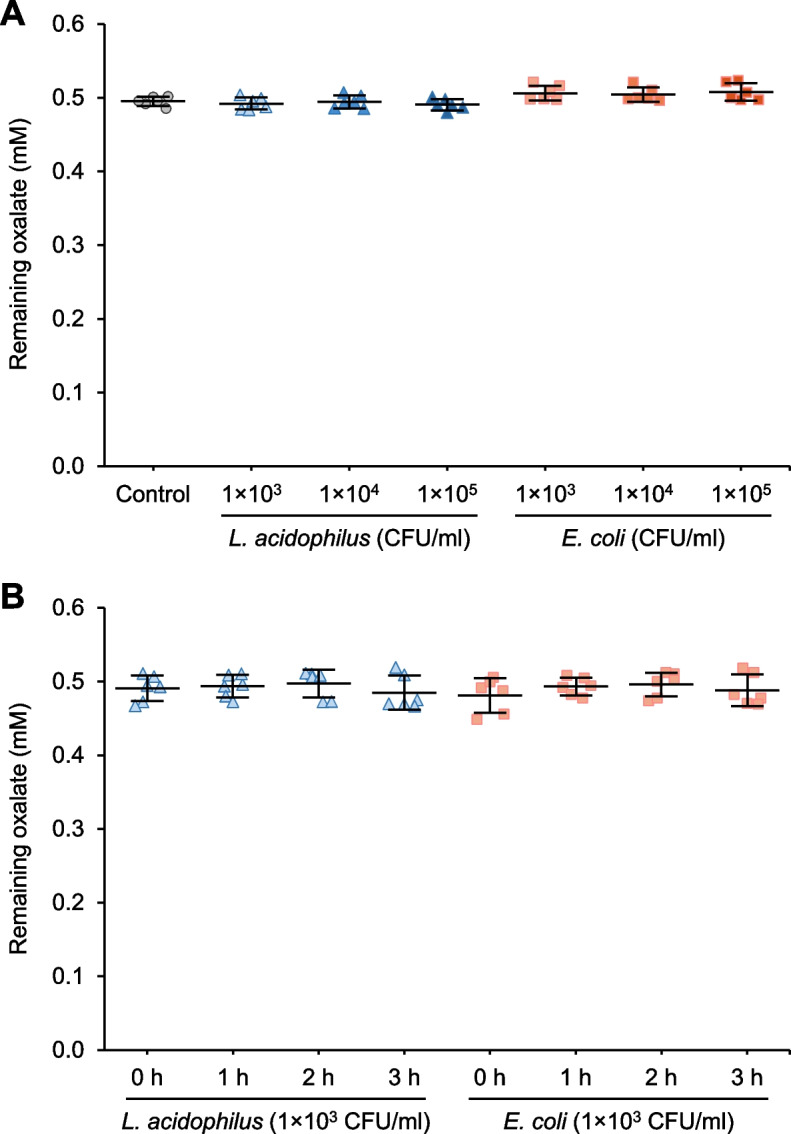
Analysis of oxalate consumption by bacteria. **A** Oxalate consumption assay was performed without (control) or with 1 × 10^3^, 1 × 10^4^ or 1 × 10^5^ CFU/ml of *L. acidophilus* or *E. coli* for 1 h. **B** Oxalate consumption assay was performed without (control) or with 1 × 10^3^ CFU/ml of *L. acidophilus* or *E. coli* for 1, 2 or 3 h. The remaining oxalate concentration in the supernatant was determined by measuring its absorbance at λ214 nm using a UV–visible spectrophotometer compared with a standard curve. Quantitative data were obtained from three independent experiments using independent biological replicates. The error bar represents SD. There were no significant differences observed among groups

To investigate mechanisms underlying the inhibitory effects of *L. acidophilus* on all the crystal features mentioned above, we first examined its potential effect on oxalate consumption (or degradation). First, the sensitivity of our oxalate measurement assay was determined by a standard method [38–40] applied to many recent works [41–43]. Using known oxalate concentrations at 0, 0.1, 0.2, 0.3, 0.4, 0.5, 0.6, 0.7 and 0.8 mM to construct the standard curve, the sensitivity (k_A_) of the assay at individual standards was at 0.3892 – 0.3976 A.U./mM (Supplementary Figure S2). Since the constructed standard curve was considerably linear (with the coefficient of determination or *R*^2^ = 0.9999), the k_A_ could be assumed at 0.3964 A.U./mM for the whole assay using this series of multiple standards (Supplementary Figure S2). The oxalate consumption assay revealed that neither *L. acidophilus* nor *E. coli* at 1 × 10^3^, 1 × 10^4^ or 1 × 10^5^ CFU/ml significantly reduced the remaining oxalate level in the crystallization buffer, indicating that there was no oxalate consumption/degradation after 1-h incubation (Fig. 5A). Although the incubation time was extended to 3 h, neither *L. acidophilus* nor *E. coli* at 1 × 10^3^ CFU/ml significantly reduced the remaining oxalate level (Fig. 5B). However, the positive result was seen when *L. acidophilus* increased to 1 × 10^7^ CFU/ml, which caused a significant reduction of the remaining oxalate after 1-h incubation (Supplementary Figure S3). Nevertheless, it should be noted that such (too high) bacterial concentration (1 × 10^7^ CFU/ml) is far beyond the physiologic range of commensal bacteria in normal human urine [16, 46, 47].

### Binding of bacteria to CaOx crystals

**Fig. 6:**
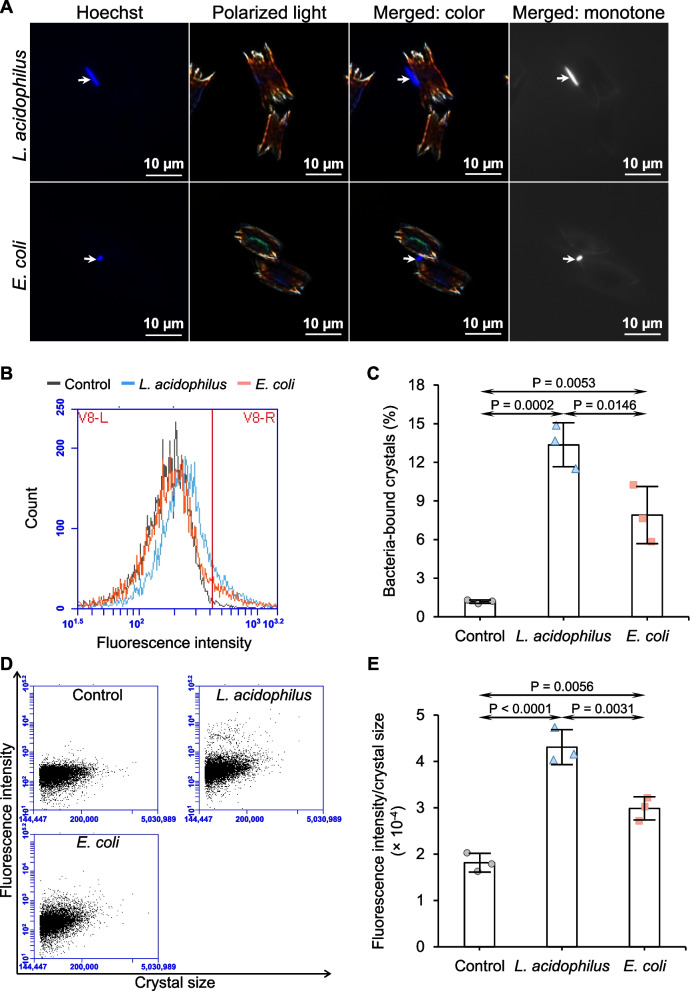
Analysis of bacteria-crystal binding. Bacteria-crystal binding assay was performed to determine the crystal-binding capability of *L. acidophilus* or *E. coli*. **A** Qualitative analysis by fluorescence staining of bacterial DNA using Hoechst dye (the bacteria are in blue and indicated with white arrows). **B** Quantitative analysis by flow cytometry using propidium iodide to stain bacterial DNA. The crystals without bacterial incubation served as the control. **C** Percentage of the bacteria-bound crystals was then quantified. **D** Scatter plot of fluorescence intensity and crystal size. **E** Fluorescence intensity/crystal size ratio was then quantified. Quantitative data were obtained from three independent experiments using independent biological replicates. The error bar represents SD, and only significant *P* values are indicated

As *L. acidophilus* did not significantly degrade oxalate under our experimental conditions, we hypothesized that the protective effects of *L. acidophilus* on CaOx stone development might be from its ability to directly bind to CaOx crystals. We, therefore, qualitatively and quantitatively analyzed the binding of *L. acidophilus* and *E. coli* to CaOx crystals. Qualitative analysis by fluorescence (Hoechst) staining of the bacterial DNA revealed that both *L. acidophilus* and *E. coli* could bind to CaOx crystals (as indicated by the blue rod on the crystal surfaces) (Fig. 6A). Quantitative analysis of the bacteria-bound CaOx crystals was then performed by flow cytometry (Fig. 6B-E). The data showed that percentages of bacteria-bound crystals by both *L. acidophilus* and *E. coli* were significantly greater than that of the control (Fig. 6B and C). Moreover, the percentage of *L. acidophilus*-bound crystals was significantly greater than that of *E. coli*-bound crystals (Fig. 6B and C). In concordance, the fluorescence intensity/crystal size ratio in the presence of *L. acidophilus* or *E. coli* was significantly greater than that of the control (Fig. 6D and E). Additionally, the fluorescence intensity of *L. acidophilus*/crystal size was significantly greater than the fluorescence intensity of *E. coli*/crystal size (Fig. 6D and E).

### The presence of S-layer protein and flagella on bacterial surface

**Fig. 7:**
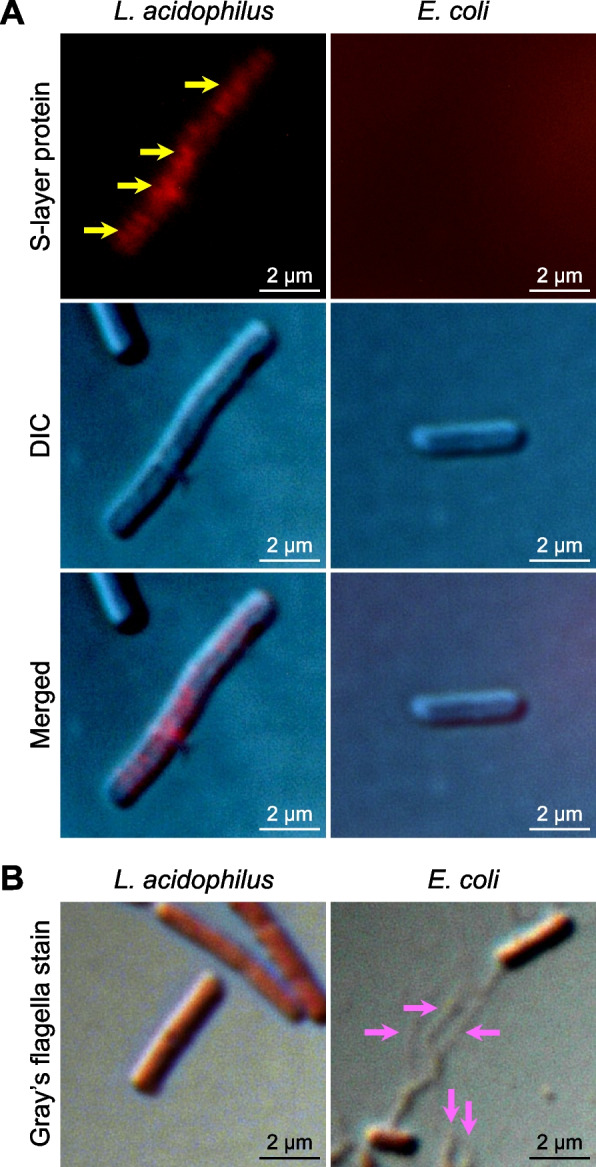
Detection of S-layer protein and flagella on bacterial surface. **A** Immunofluorescence staining was performed to detect S-layer protein (indicated by yellow arrows) on bacterial surface. **B** Gray’s flagella staining was performed to detect bacterial flagella (indicated by pink arrows)

Although both *L. acidophilus* and *E. coli* bound to CaOx crystals, they had opposite effects on CaOx stone development (hampered by *L. acidophilus* but induced by *E. coli*). As such, we hypothesized that *L. acidophilus* and *E. coli* possessed different adhesive surface components that affected CaOx differently. Hence, S-layer protein and flagella, both of which are the bacterial surface components reported with adhesive properties [45, 48–53], were tested on *L. acidophilus* and *E. coli*. Immunofluorescence staining of S-layer protein revealed that this surface protein was present only on *L. acidophilus* (as indicated by yellow arrows in Fig. 7A). On the other hand, Gray’s flagella staining showed that flagella were present only on the surface of *E. coli* (as indicated by pink arrows in Fig. 7B).

### Effects of S-layer-removed *L. acidophilus* and flagella-removed *E. coli* on CaOx stone development

**Fig. 8:**
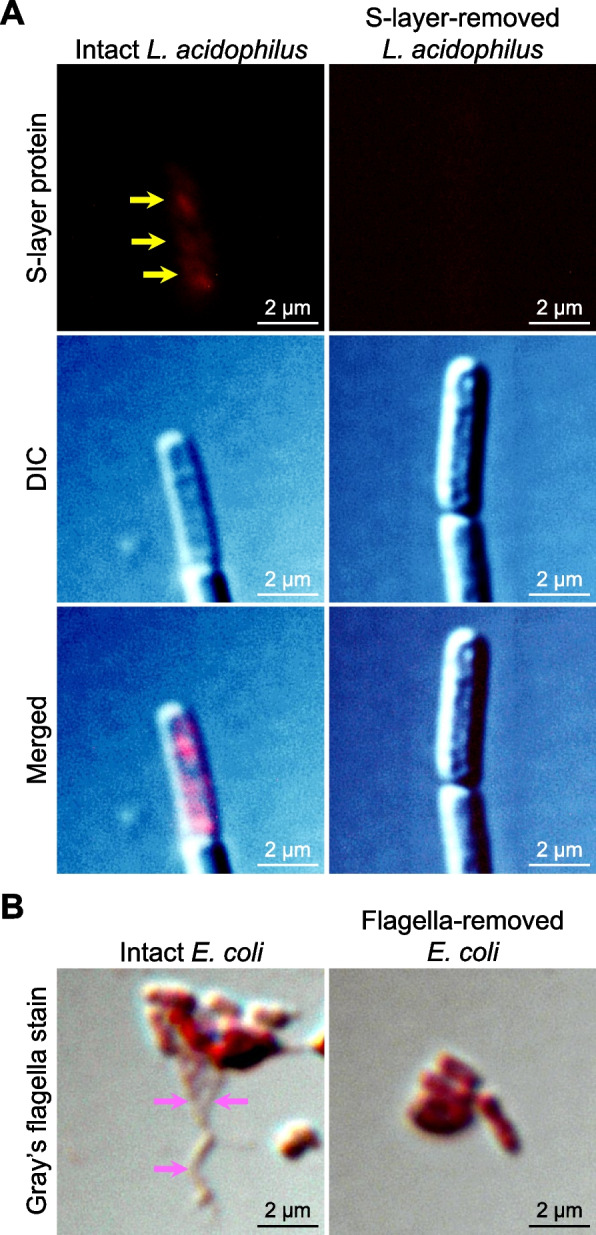
Confirmation of S-layer protein and flagella removal from bacterial surfaces. **A** *L. acidophilus* was incubated with 5 M LiCl for 2 h to remove S-layer protein. Both intact (untreated or control) and S-layer-removed *L. acidophilus* were then subjected to immunofluorescence staining to detect S-layer protein (indicated by yellow arrows) on bacterial surface. **B** *E. coli* was acidified with acetic acid to make the pH at 4.5 and incubated for 45 s to remove flagella. Both intact (untreated or control) and flagella-removed *E. coli* were then subjected to Gray’s flagella staining to detect the flagella (indicated by pink arrows)

**Fig. 9:**
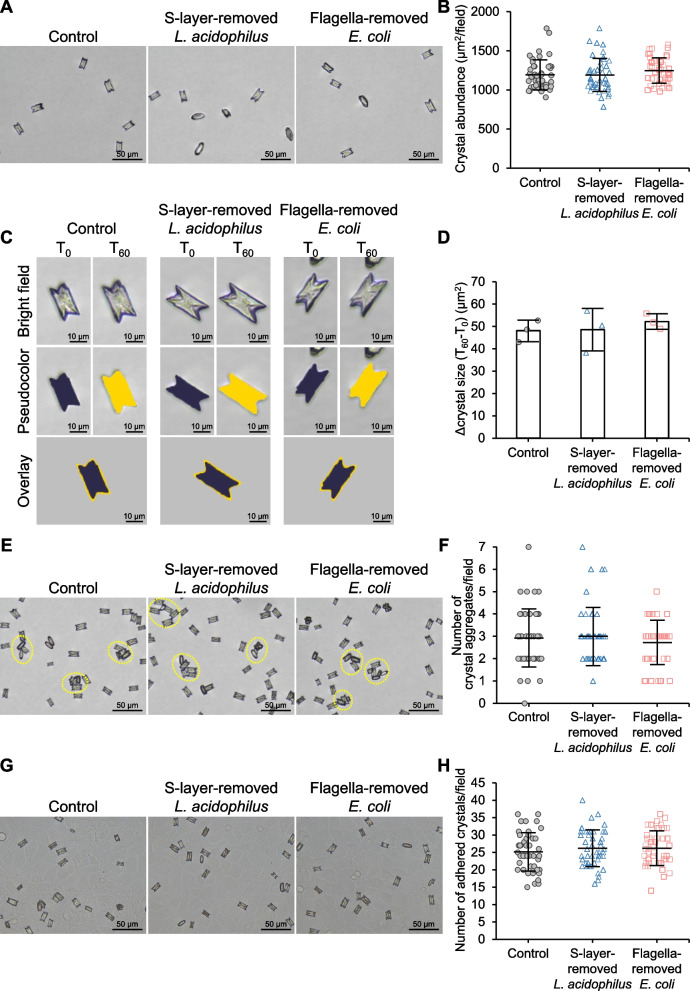
Effects of S-layer-removed *L. acidophilus* and flagella-removed *E. coli* on CaOx crystals. All the crystal assays were performed without (control) or with 1 × 10^3^ CFU/ml of S-layer-removed *L. acidophilus* or flagella-removed *E. coli*. **A** Representative images of CaOx crystals that were generated under different conditions. **B** Crystal abundance was calculated using *Formula I* (see Materials and methods) from ≥ 15 random fields in each sample. **C** Representative images of CaOx crystals at T_0_ and T_60_ that were generated under different conditions. Pseudocolor and overlay views were created to enhance the visualization of differential sizes of crystals at T_0_ and T_60_. **D** Δ crystal size representing the growth of crystals was calculated using *Formula II* (see Materials and methods) from ≥ 100 crystals in ≥ 10 random fields in each sample. **E** Representative images of CaOx aggregates (marked with dotted circles) that were generated under different conditions. **F** The aggregate number was counted from ≥ 15 random fields in each sample. **G** Representative images of adherent CaOx crystals that remained on the cell monolayer under different conditions. **H** Number of the adhered crystals was counted from ≥ 15 random fields in each sample. All quantitative data were obtained from three independent experiments using independent biological replicates. The error bar represents SD. There were no significant differences observed among groups in all assays

To investigate whether S-layer protein and flagella were responsible for CaOx stone inhibitory and promoting effects of *L. acidophilus* and *E. coli*, respectively, S-layer protein and flagella were removed from the bacteria. The absence of S-layer protein and flagella was clearly demonstrated in the S-layer-removed *L. acidophilus* (Fig. 8A) and flagella-removed *E. coli* (Fig. 8B), respectively, indicating the successfulness of the removal of S-layer protein and flagella. The S-layer-removed *L. acidophilus* and flagella-removed *E. coli* at an equal concentration (1 × 10^3^ CFU/ml, which was identical to that used for the intact bacteria) were subjected to all the crystal assays tested for the intact bacteria. The data showed that these S-layer-removed *L. acidophilus* and flagella-removed *E. coli* had no significant effects on crystallization (Fig. 9A and B), growth (Fig. 9C and D), aggregation (Fig. 9E and F) and cell-adhesive ability of CaOx (Fig. 9G and H). These data strengthened a hypothesis that the inhibitory effects of *L. acidophilus* and the promoting effects of *E. coli* on CaOx stone development were most likely from S-layer protein and flagella, respectively.

## Discussion

The urinary tract of healthy individuals is known to harbor several bacterial genera, e.g., *Bifidobacterium*, *Lactobacillus*, *Prevotella*, *Staphylococcus*, and *Streptococcus* [15, 19]. Alterations in such bacterial community or urinary microbiome have been reported in many kidney diseases, including KSD [54–56]. Several bacteria, including *E. coli, Enterococcus* spp. and *Klebsiella* spp., are detected in stone matrices and urine of stone patients [9, 10, 56]. Furthermore, *E. coli* and *K. pneumoniae* have been reported to exert stimulatory effects on CaOx stone development involving crystallization, crystal growth and crystal aggregation [11, 12].

Herein, we examined the effects of *E. coli* (ATCC 25922™ or strain Seattle 1946) on CaOx crystals compared with *L. acidophilus* (ATCC 314™). *E. coli* strain Seattle 1946 was clinically isolated and considered a pathogenic strain (https://bacdive.dsmz.de/strain/4427). Although there was no clear evidence demonstrating the association of *E. coli* strain Seattle 1946 with KSD, previous studies have clearly shown its effects on CaOx stone development in vitro [11, 12]. In agreement with the previous reports using this strain of *E. coli* [11, 12], our present study showed that *E. coli* strain Seattle 1946 enhanced CaOx crystal growth and aggregation. However, under the experimental conditions of this work, *E. coli* did not show a significant effect on CaOx crystallization. These disparate results were most likely from the different concentrations of *E. coli* being used. The previous reports [11, 12] used 10^5^ CFU/ml of *E. coli* (which is considered an infected condition and is one of the criteria to diagnose urinary tract infection [46, 47]). However, the present study used a much lower concentration of *E. coli* (1 × 10^3^ CFU/ml) to simulate a physiologic (normal or non-infected) state of the urinary tract with commensal bacteria [16, 46, 47].

In contrast to the bacteria that are highly abundant in the urine of stone patients, many commensal bacteria, especially *Lactobacillus* spp., are less abundant in the urine of stone patients [8]. Also, the abundance of *Lactobacillus* spp. in kidney stone matrices is extremely low [9]. Besides, metagenomic analysis of urinary microbiome has revealed that *Lactobacillus*-derived genes are enriched in healthy individuals [18]. Hence, *Lactobacillus* spp. have been suggested to play protective roles in KSD [7]. However, functions of *Lactobacillus* spp. in the urinary tract related to stone development had not yet been elucidated. The present study is the first one to demonstrate the direct effects of *L. acidophilus* on various steps of CaOx stone development. The results have shown that *L. acidophilus* significantly reduced CaOx crystallization, enlargement, aggregation and adhesion to collecting duct epithelial cell surfaces.

Usually, the urinary microbiome is characterized from bladder urine and pelvis urine [13, 23, 24, 57]. The inner medullary collecting duct is the area close to the renal pelvis. Therefore, we used mouse inner medullary collecting duct (mIMCD-3) cells instead of other renal cells in this study as they can better simulate the in vivo scenario where the commensal bacteria in the pelvis closely contact the collecting duct cells.

Indeed, *L. acidophilus* has been documented with its oxalate-degrading ability in the gastrointestinal tract [58–60]. Therefore, we first hypothesized that the preventive effects of *L. acidophilus* against CaOx crystals might be from its oxalate-degrading ability. However, under our experimental conditions, *L. acidophilus* was unable to degrade oxalate as indicated by no significant reduction of oxalate level after the oxalate consumption assay. Such discordant results were probably because of different experimental conditions of the oxalate consumption assay between previous reports and this study. In previous reports [58–60], *L. acidophilus* was exposed to oxalate dissolved in MRS broth, which is a common growth medium for bacteria. On the other hand, in our present study, *L. acidophilus* was exposed to oxalate dissolved in the crystallization buffer, which contained no carbon source except for oxalate.

It has been demonstrated that *Bacillus subtilis*, *Deinococcus* spp. and *E. coli* can survive under carbon-starvation conditions [61, 62]. However, the growth rate of *B. subtilis* under the carbon-starvation condition is extremely low (with a 4-day doubling time) [62]. *L. acidophilus* cultured in a medium without glucose supplementation exhibits a much slower growth than that cultured in a medium supplemented with glucose [63]. Additionally, *L. acidophilus* grows slower in a non-glucose medium supplemented with oxalate than in a glucose-containing medium [63], suggesting that oxalate is not the main carbon source used by *L. acidophilus* for its growth and survival. In this present study, *L. acidophilus* was exposed to oxalate dissolved in the crystallization buffer during the oxalate-consumption assay. Without other carbon sources and nutrients essentially required for bacterial survival, it might be hard for *L. acidophilus* to survive and maintain its functions by consuming only oxalate.

Although *Lactobacillus* spp. are categorized as the generalist oxalotrophs (as they use oxalate as an alternative carbon source [64]), oxalate (particularly at high concentrations) may have some toxic effects on some *Lactobacillus* spp. [63, 65]. For instance, 20 mM Na_2_C_2_O_4_ reduces the growth of *L. johnsonii* La1, *L. reuteri* Bio, and *L. casei* Lbc496 [65], and 35 mM (NH_4_)_2_C_2_O_4_ reduces the growth of *L. acidophilus* NCFM [63]. *Lactobacillus* spp. then degrade oxalate to further grow and/or survive. It should be noted that the concentration of Na_2_C_2_O_4_ used in our present study was quite low (0.5 mM) and the incubation time was relatively brief (only 1 − 3 h) (to simulate the effects of the intact bacteria in all CaOx crystal assays, which were done within only 1 h). Therefore, the undetectable oxalate-degrading activity of *L. acidophilus* in this study might be from these two factors. A much longer incubation and much higher concentration of Na_2_C_2_O_4_ may be required to observe the oxalate-degrading ability of *L. acidophilus*. In addition, the low or physiologic concentration of *L. acidophilus* used in our present study (1 × 10^3^ CFU/ml) might be below its capability to demonstrate a significant reduction of the remaining oxalate level. As expected, such positive results could be achieved when the concentration of *L. acidophilus* went up to 1 × 10^7^ CFU/ml. However, this extremely high concentration of bacteria is not physiologic and exists only when infection occurs [46, 47].

Since the oxalate-degrading ability of *L. acidophilus* was not detected in our experimental settings using a physiologic concentration of commensal bacteria, we further explored another mechanism that was more likely to underly the protective effects of *L. acidophilus* against CaOx stone development. Several *Lactobacillus* spp.*,* such as *L. fermentum*, *L. gasseri*, and *L. plantarum*, have been reported with adhesive properties to bind to mammalian cell surfaces and to form aggregates with other bacteria [66–68]. Accordingly, we hypothesized that *L. acidophilus* would interfere with CaOx stone development processes through its ability to bind to CaOx crystals. The data clearly exhibited that *L. acidophilus* was able to bind to CaOx crystals. However, not only *L. acidophilus* but also *E. coli*, a bacterium that promoted CaOx stone development, bound to CaOx crystals. The binding of *E. coli* to CaOx crystals demonstrated herein was consistent with that observed in the previous report showing the binding of *E. coli* to the aggregates of CaOx crystals [69]. Surprisingly, we observed a higher percentage of the *L. acidophilus*-bound CaOx crystals than that of the *E. coli*-bound CaOx crystals, suggesting the more potent crystal-binding capability of *L. acidophilus*.

Because both *L. acidophilus* and *E. coli* could bind to CaOx but exerted contradictory activities on the crystals, we then hypothesized that they had different adhesive molecules on their surfaces that had unique modulatory effects on the crystals. S-layer protein is one of the common surface components and abundant proteins localized on the cell wall of some bacteria [70–73]. Genes encoding S-layer proteins have been identified in various *Lactobacillus* spp. [74]. The S-layer plays a role in adhesive capability of bacteria, as deletion of genes encoding S-layer-associated proteins reduces the adhesion of *L. acidophilus* NCFM to Caco-2 cells and extracellular matrix proteins [48]. In addition, removal of the S-layer from *L. casei*, *L. plantarum*, and *L. coryniformis* decreases the binding of bacteria to intestinal epithelium [45]. Moreover, S-layer proteins isolated from *Clostridium difficile* can bind to CHO-K1 ovary cell membrane [49].

Interestingly, it has been documented that S-layer proteins can bind some divalent ions [74]. For example, S-layer proteins of *L. kefir* can bind Pb^2+^ [75]. S-layer proteins of *Caulobacter crescentus* and *Lysinibacillus sphaericus* bind Ca^2+^ and Mg^2+^, which play roles in assembling and stabilizing the S-layer structure [76–78]. Accordingly, we hypothesized that S-layer protein would be detected on the surface of *L. acidophilus*, not *E. coli*, and get involved in CaOx crystal inhibition observed in our present study. The immunofluorescence study confirmed that the S-layer protein was detected only on *L. acidophilus* surface, not on *E. coli* surface.

Concerning bacterial surface components that might play roles in promoting CaOx stone development, it has been documented that some bacterial appendages, e.g., fimbria, flagellum and pilus, involve bacterial adhesion to materials and surfaces of other cells [50–53]. Flagella isolated from *E. coli* have been previously reported to induce CaOx crystallization, growth and aggregation [12]. Moreover, deflagellated *E. coli* lose its ability to promote CaOx stone formation [12]. Interestingly, only a few of *Lactobacillus* spp. are motile, and *L. acidophilus* is not among these [79, 80]. We thus hypothesized that flagellum was absent on *L. acidophilus* surface but was the key surface component on *E. coli* surface that determined the enhancing activity of *E. coli* on CaOx crystal growth and aggregation. Gray’s flagella staining confirmed that flagella were detected on *E. coli* surface, but not on *L. acidophilus* surface.

To address our hypotheses on the inhibitory activities of *L. acidophilus* S-layer protein and promoting activities of *E. coli* flagella on CaOx stone development, we examined the effects of the S-layer-removed *L. acidophilus* and flagella-removed *E. coli* on CaOx crystals. As expected, the data revealed that the inhibitory and promoting effects of *L. acidophilus* and *E. coli*, respectively, on CaOx crystals were completely abolished when S-layer protein and flagella were removed from the bacteria.

Although promising, some limitations of our present study should be noted. First, the modulatory effects of bacteria on KSD are not limited only to *L. acidophilus* and *E. coli* strains used in our present study. As discussed earlier, there are other *Lactobacilli* spp. identified in the urinary microbiome that have been reported to be associated with KSD. Therefore, the protective roles of other *Lactobacilli* spp. and the promoting effects of other *E. coli* strains should be also elucidated to gain the entire image of the modulatory effects of *Lactobacilli* spp. and individual *E. coli* strains on CaOx stone development. Second, although our oxalate consumption assay could demonstrate the positive result at a high bacterial concentration (which is non-physiologic and irrational for investigating the effects of commensal bacteria in normal urine), employing a more sensitive technique to measure the remaining oxalate would be beneficial to detect oxalate consumption at a trivial amount by commensal bacteria under a physiologic setting. Finally, our present study was done entirely in vitro. Validation in an in vivo setting would be ideal for translating this information to clinical implications.

## Conclusions

The present study has provided, for the first time, evidence to demonstrate the direct effects of *L. acidophilus* to prevent CaOx stone development. *L. acidophilus* hampers crystallization, growth, aggregation and cell-adhesive ability of CaOx. By contrast, *E. coli* induces CaOx stone development by promoting CaOx growth and aggregation. Removal of *L. acidophilus* S-layer protein and *E. coli* flagella completely abolished the inhibitory and promoting effects of *L. acidophilus* and *E. coli*, respectively. These data implicate that their contradictory effects are most likely from differential surface components (i.e., S-layer protein on *L. acidophilus* and flagella on *E. coli*) not from oxalate-degrading ability.

## Supplementary Information

Supplementary Material 1.

## Data Availability

All data generated or analyzed during this study are included in this published article and are also available from the corresponding author on reasonable request.
