# Supplementary material for: The direct inhibitory effects of Lactobacillus acidophilus, a commensal urinary bacterium, on calcium oxalate stone development
Source: Microbiome. 2024 Sep 17;12:175. doi: 10.1186/s40168-024-01877-y (PMC11406782; doi:10.1186/s40168-024-01877-y)
Supplement: Supplementary file 2 — Supplementary Material 1. [file 40168_2024_1877_MOESM1_ESM.pdf]

SUPPORTING INFORMATION

The direct inhibitory effects of *Lactobacillus acidophilus*, a commensal urinary bacterium, on calcium oxalate stone development

Chadanat Noonin, Anantaya Putpim, and Visith Thongboonkerd*

* Correspondence to: *thongboonkerd@dr.com (or) vthongbo@yahoo.com*

Figure S1: FTIR analysis of our CaOx crystals. The CaOx crystals generated in this study were analyzed for their chemical composition using FTIR spectroscopy. The data showed that our CaOx crystals (B) perfectly matched with CaOx monohydrate in the reference database (A).

Figure S2: Sensitivity of oxalate measurement. Standard curve of $Na_2C_2O_4$ at 0, 0.1, 0.2, 0.3, 0.4, 0.5, 0.6, 0.7 and 0.8 mM in crystallization buffer was constructed. The sensitivity (k_A) of this assay at each standard point was calculated using a formula illustrated above. Additionally, the linearity of this standard curve was achieved with a coefficient of determination (R^2) = 0.9999. Therefore, the k_A of this assay using the mentioned series of multiple standards could be assumed at 0.3964 A.U./mM.

Figure S3: Analysis of oxalate consumption by high concentrations of *L. acidophilus*. Oxalate consumption assay was performed without (control) or with 1×10^6 or 1×10^7 CFU/ml of *L. acidophilus* for 1 h. The remaining oxalate concentration in the supernatant was determined by measuring its absorbance at λ214 nm using a UV-visible spectrophotometer compared with a standard curve. Quantitative data were obtained from three independent experiments using independent biological replicates. The error bar represents SD, and only significant P values are indicated.

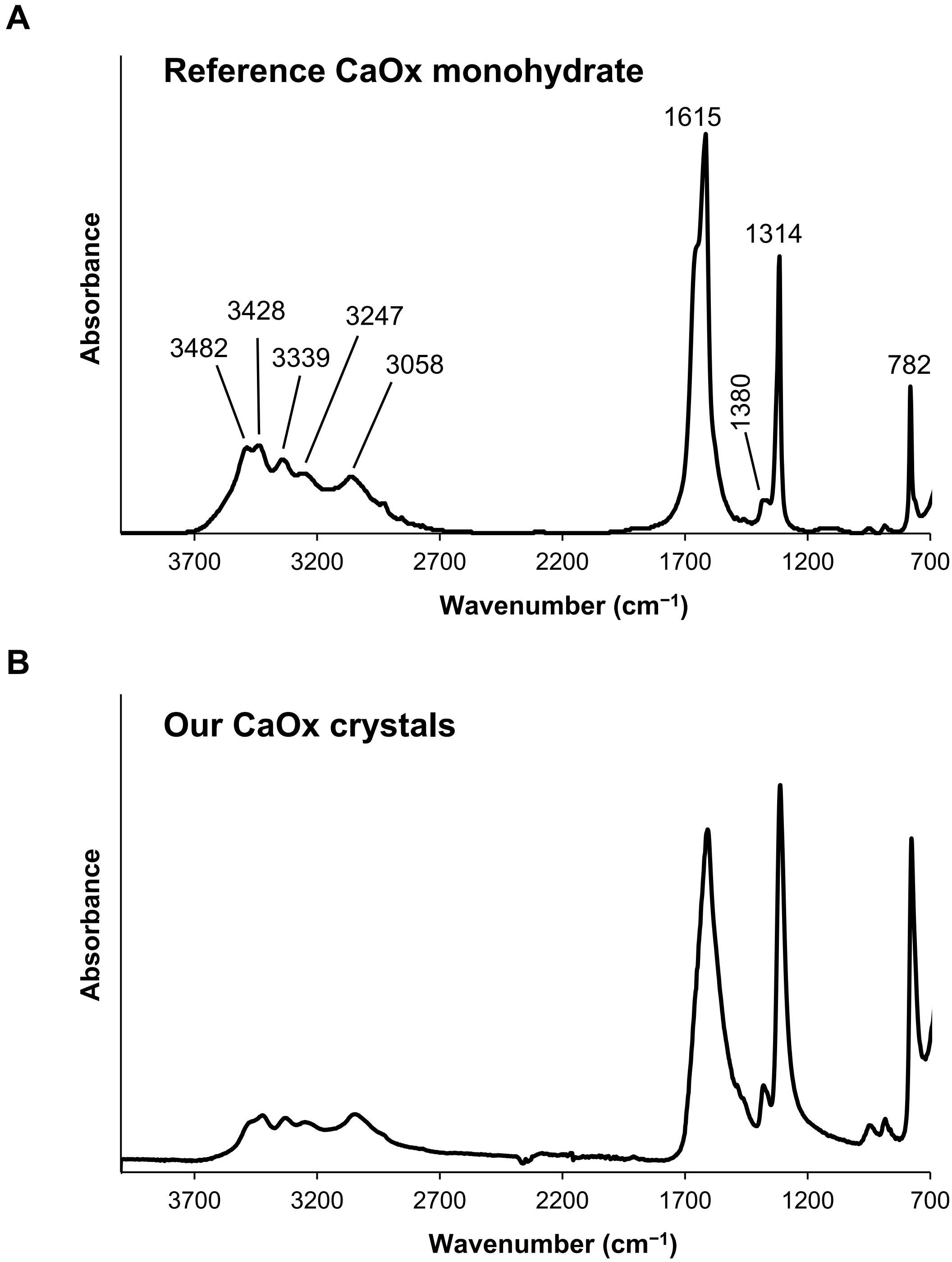

Figure S1: FTIR analysis of our CaOx crystals. The CaOx crystals generated in this study were analyzed for their chemical composition using FTIR spectroscopy. The data showed that our CaOx crystals (B) perfectly matched with CaOx monohydrate in the reference database (A).

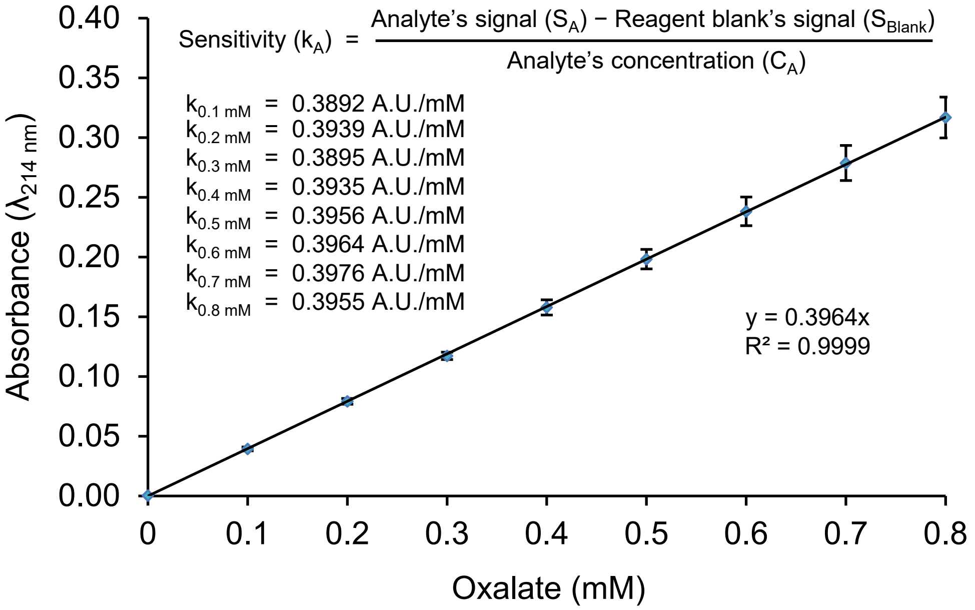

Figure S2: Sensitivity of oxalate measurement. Standard curve of $Na_2C_2O_4$ at 0, 0.1, 0.2, 0.3, 0.4, 0.5, 0.6, 0.7 and 0.8 mM in crystallization buffer was constructed. The sensitivity (k_A) of this assay at each standard point was calculated using a formula illustrated above. Additionally, the linearity of this standard curve was achieved with a coefficient of determination (R^2) = 0.9999. Therefore, the k_A of the whole assay using the mentioned series of multiple standards could be assumed at 0.3964 A.U./mM.

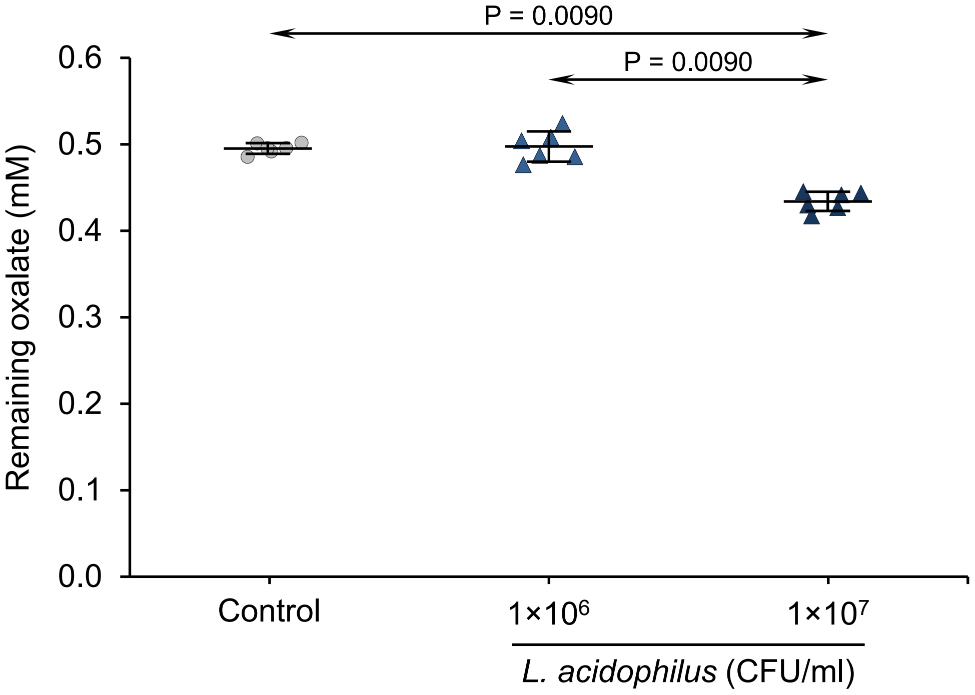

Figure S3: Analysis of oxalate consumption by high concentrations of *L. acidophilus*. Oxalate consumption assay was performed without (control) or with 1 $\times 10^6$ or 1 $\times 10^7$ CFU/ml of *L. acidophilus* for 1 h. The remaining oxalate concentration in the supernatant was determined by measuring its absorbance at λ214 nm using a UV-visible spectrophotometer compared with a standard curve. Quantitative data were obtained from three independent experiments using independent biological replicates. The error bar represents SD, and only significant P values are indicated.
